# EPO and TMBIM3/GRINA Promote the Activation of the Adaptive Arm and Counteract the Terminal Arm of the Unfolded Protein Response after Murine Transient Cerebral Ischemia

**DOI:** 10.3390/ijms20215421

**Published:** 2019-10-31

**Authors:** Pardes Habib, Ann-Sophie Stamm, Joerg B. Schulz, Arno Reich, Alexander Slowik, Sandro Capellmann, Michael Huber, Thomas Wilhelm

**Affiliations:** 1 Department of Neurology, Medical Faculty, RWTH Aachen University, 52074 Aachen, Germany; ann-sophie.stamm@rwth-aachen.de (A.-S.S.); jschulz@ukaachen.de (J.B.S.); areich@ukaachen.de (A.R.); 2 JARA-BRAIN Institute Molecular Neuroscience and Neuroimaging, Forschungszentrum Jülich GmbAnd RWTH Aachen University, 52074 Aachen, Germany; 3 Institute of Neuroanatomy, Medical Faculty, RWTH Aachen University, 52074 Aachen, Germany; aslowik@ukaachen.de; 4 Institute of Biochemistry and Molecular Immunology, Medical Faculty, RWTH Aachen University, 52074 Aachen, Germany; scapellmann@ukaachen.de (S.C.); mhuber@ukaachen.de (M.H.); twilhelm@ukaachen.de (T.W.)

**Keywords:** UPR, GSK-2606414, STF-083010, PERK, IRE1α, stroke, GRINA, neuroprotection, EPO

## Abstract

Ischemic stroke is known to cause the accumulation of misfolded proteins and loss of calcium homeostasis leading to impairment of endoplasmic reticulum (ER) function. The unfolded protein response (UPR) is an ER-located and cytoprotective pathway that aims to resolve ER stress. Transmembrane BAX inhibitor-1 motif-containing (TMBIM) protein family member TMBIM3/GRINA is highly expressed in the brain and mostly located at the ER membrane suppressing ER calcium release by inositol-1,4,5-trisphosphate receptors. GRINA confers neuroprotection and is regulated by erythropoietin (EPO) after murine cerebral ischemia. However, the role of GRINA and the impact of EPO treatment on the post-ischemic UPR have not been elucidated yet. We subjected GRINA-deficient (*Grina^−/−^*) and wildtype mice to transient (30 min) middle cerebral artery occlusion (tMCAo) followed by 6 h or 72 h of reperfusion. We administered EPO or saline 0, 24 and 48 h after tMCAo/sham surgery. Oxygen–glucose deprivation (OGD) and pharmacological stimulation of the UPR using Tunicamycin and Thapsigargin were carried out in primary murine cortical mixed cell cultures. Treatment with the PERK-inhibitor GSK-2606414, IRE1a-RNase-inhibitor STF-083010 and EPO was performed 1 h prior to either 1 h, 2 h or 3 h of OGD. We found earlier and larger infarct demarcations in *Grina^−/−^* mice compared to wildtype mice, which was accompanied by a worse neurological outcome and an abolishment of EPO-mediated neuroprotection after ischemic stroke. In addition, GRINA-deficiency increased apoptosis and the activation of the corresponding PERK arm of the UPR after stroke. EPO enhanced the post-ischemic activation of pro-survival IRE1a and counteracted the pro-apoptotic PERK branch of the UPR. Both EPO and the PERK-inhibitor GSK-2606414 reduced cell death and regulated *Grina* mRNA levels after OGD. In conclusion, GRINA plays a crucial role in post-ischemic UPR and the use of both GSK-2606414 and EPO might lead to neuroprotection.

## 1. Introduction

Stroke occurs due to an insufficient blood perfusion to the brain and is a major cause of adult disability and death in the western world [1,2]. In the brain region with the most severely restricted blood flow, termed the ischemic core, necrotic cell death occurs within minutes. In the adjacent penumbra area, however, collateral blood flow can buffer the full effects of ischemia; hence, delayed cell death occurs through apoptosis. This region might be amenable to therapeutic interventions. The great variance in brain damage and neurological impairments resulting from stroke are mainly caused by the respective ischemic vulnerability of various brain regions and their different cell types. The current therapy of ischemic stroke includes reperfusion modalities such as thrombolysis by the serine protease, recombinant tissue plasminogen activator (rtPA) and mechanical endo-vascular thrombectomy. Approaches of pharmacological cell protection have not been successful so far [3,4]. To improve stroke outcome, new pharmacological approaches must be considered, such as boosting endogenous pro-survival pathways. Among them, the unfolded protein response (UPR) is a promising cellular mechanism, since the UPR maintains and restores endoplasmic reticulum (ER) function, which is critical for the survival of stressed cells in general and of hypoxic cells in particular [5,6,7,8].

Amongst others, one important function of the ER is the adequate translation and folding of transmembrane and secretory proteins. Accumulation of misfolded proteins in the ER triggers a complex cellular stress response, the UPR [9]. This signaling system is dynamic and highly regulated and is initiated and activated via three sensors depending on the type, duration and intensity of the stimulus: IRE1α (inositol-requiring enzyme 1α), PERK (protein kinase R-like ER kinase) and ATF6 (activating transcription factor 6). Upon activation, IRE1α cleaves the mRNA encoding for the X-box binding protein (XBP1), removing a 26 nucleotide intron and prompting the translation of spliced XBP1 (XBP1s). The latter is a transcription factor and controls the expression of a large number of ER chaperones and proteins involved in ER-associated degradation (ERAD) including, for instance, DnaJ Heat Shock Protein Family (Hsp40) Member B9 (DNAJB9) [10,11,12]. In addition, the IRE1α RNase domain directly degrades diverse mRNAs and microRNAs through a process termed “Regulated IRE1-Dependent Decay” (RIDD), regulating inflammation and apoptosis amongst others [13,14,15,16]. Activated PERK mediates protein translation shutdown via phosphorylation of the eukaryotic initiation factor 2α (P-eIF2α), which also favors selective translation of certain mRNAs encoding particular proteins, including the transcription factor ATF4, which is involved in the switch towards the terminal, apoptotic arm of the UPR [16,17]. ATF6, upon ER stress, trans-locates to the Golgi apparatus where it is cleaved by membrane-bound site-1 and site-2 proteases resulting in the release of its cytoplasmic N-terminal domain that directs the expression of genes encoding ERAD components and ER chaperones [16,18]. Several studies have proposed that brain ischemia impairs ER function and activates the UPR, since the expression of target genes of the three UPR branches, including *Grp78*, *Pdia4* and *Ddit3/Chop* were found up-regulated after ischemic stroke [19,20,21,22,23]. Furthermore, activation of *Xbp1* mRNA splicing and post-ischemic shutdown of translation resulting from phosphorylation of eIF2α were evident after cerebral ischemia, implying a major role of the IRE1α and the PERK arm of the UPR in stroke pathology [24,25,26,27,28]. Although we know that ischemic stroke impairs ER function and activates the UPR, we do not yet understand how the individual UPR branches define the outcome and function of post-ischemic cortical cells, nor, which UPR branch or branches play a predominant role for stroke outcome.

The anti-apoptotic transmembrane BAX Inhibitor-1 motif-containing (TMBIM) protein family member TMBIM3/GRINA has been reported to be upregulated in cellular and animal models of ER stress [29]. This 38 kDa protein is conserved among different species and highly abundant in the brain. Furthermore, GRINA does not only seem to be equally expressed in cortex, striatum, cerebellum and hindbrain, but also shows similar expression levels in neurons, astrocytes, microglia and oligodendrocytes on a cellular level. [30,31,32,33]. In addition, gene expression of TMBIM3/GRINA is dysregulated in brains of patients with major depression [34] and in various cancers [35]. GRINA-deficiency is not lethal in fruit flies [29] and does not show a pathological phenotype in mice, consistent with the knockout of other TMBIM family members (FAIM2, TMBIM1 and TMBIM6) [30,33,36,37]. Mostly located at the ER membrane and inhibiting ER calcium release by inositol-1,4,5-trisphosphate receptors, GRINA might have a crucial role in diminishing ER stress. We have recently demonstrated that GRINA-deficiency increased infarct volumes after murine cerebral ischemia and enhanced apoptosis rates in neurons after oxygen–glucose deprivation (OGD). In addition, GRINA conferred neuroprotection and was regulated by erythropoietin (EPO) after in vitro and in vivo ischemia [33].

The hematopoietic growth factor EPO is generated upon hypoxia and has been shown to confer beneficial effects in various neurological diseases including Parkinson’s disease [38], multiple sclerosis [39], as well as in subarachnoid hemorrhage [40], intracerebral hemorrhage [41], global ischemia [42], focal ischemia [43], neonatal hypoxia-ischemia [44], and traumatic brain injury [45]. Chiang et al. reported that ATF4 contributed to the suppression of basal and chemical hypoxia-induced (CoCl_2_; 100 μM) transcription of EPO and that downregulation of PERK expression by siRNA enhanced EPO mRNA levels in association with reduction of ATF4 in the human hepatoma cell line HepG2 [46]. However, the impact of EPO treatment and the role of GRINA on post-ischemic UPR have not been elucidated yet.

Here, we hypothesized that neuroprotection after ischemic stroke by EPO and GRINA could be mediated by the regulation of the post-ischemic UPR and in particular by the IRE1α and PERK pathways of the UPR. To test this, we subjected *Grina^−/−^* and WT mice with and without EPO administration to tMCAo and investigated the activation of the above-mentioned arms of the UPR after 6 and 72 h of reperfusion. In addition, we subjected primary cortical mixed cell cultures to OGD or pharmacologically induced ER stress and investigated the influence of GRINA and EPO on the UPR in the presence of IRE1α and PERK-inhibitors. The current study (for the first time) indicates that EPO and GRINA enhance the post-ischemic activation of pro-survival IRE1α and counteract the pro-apoptotic PERK branch of the UPR. Moreover, both EPO and PERK-inhibitor GSK-2606414 reduce cell death and regulate *Grina* transcription.

## 2. Results

### 2.1. GRINA-deficiency Leads to Early Infarct Demarcation And Larger Infarct Sizes Compared to Wildtype Littermates

**Figure 1:**
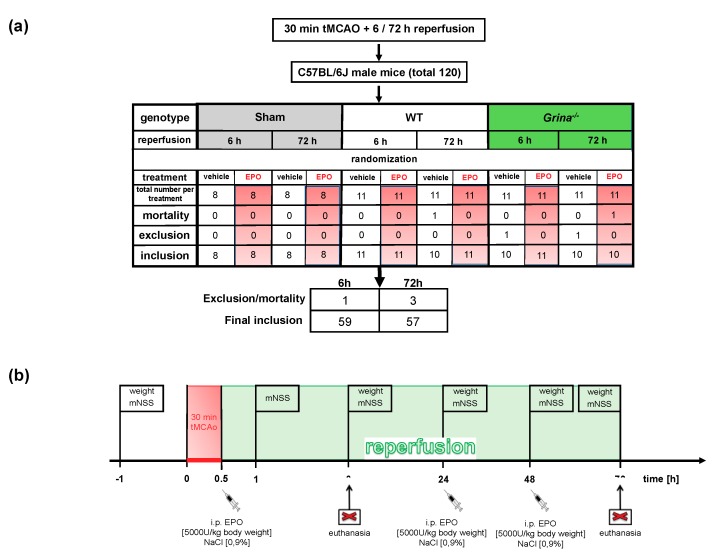
Protocol of randomized and blinded controlled trial (RCT) and study procedure (**a**) Table summarizing the number of total animals (120 mice) divided into the different experimental conditions. Mortality rate, exclusion per group and included animal for final analysis are given. Exclusion criteria: death during experiments, rCBF reduction of over 80% from baseline or no adequate increase to 60–70% of baseline after 5–10 min of reperfusion, brain haemorrhage, a weight loss of >20% of baseline, missing neurological deficits (mNSS < 5) or no evidence of infarction in TTC-staining. (**b**) Weight measurement and neurological testing (mNSS) were performed at various time-points (−1, 1, 6, 24, 48 and 72 h after stroke or sham surgery). Mice were subjected to 30 min of tMCAo or sham surgery followed by 6 or 72 h of reperfusion. EPO or saline was injected directly after surgery, 24 and 48 h after tMCAo/sham surgery.

In this preclinical randomized and blinded controlled trial (pRCT), we subjected a total of 120 male 10–12 weeks old mice to 30 min of tMCAo or sham surgery followed by 6 or 72 h of reperfusion. Three mice (one wildtype, two *Grina^−/−^* mice) died during or shortly after surgery and one *Grina^−/−^* mouse was excluded from the study due to extensive weight loss (weight loss >20% of initial weight) and was sacrificed 24 h after surgery. Hence, 116 animals (59 mice in the 6 h reperfusion period, 57 in the 72 h reperfusion period) followed the entire study protocol with 8–11 mice per genotype and treatment group (Figure 1a,b).

GRINA-deficient mice exhibited neither a spontaneous phenotype nor haematological differences compared to wildtype mice (Table 1).

**Figure 2:**
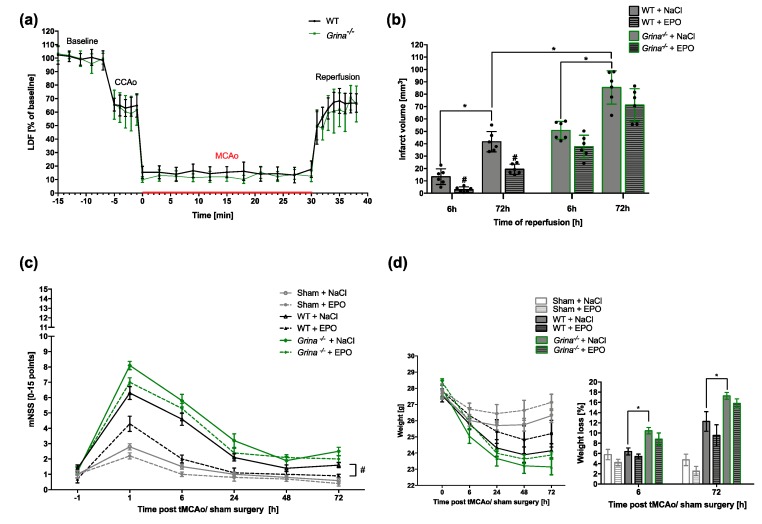
GRINA deficiency increases infarct sizes and worsens clinical outcome after murine cerebral ischemia (**a**) Ipsilateral laser doppler flowmetry was used to monitor blood perfusion after the occlusion of CCA, MCA and the early reperfusion phase. The baseline blood flow was considered as 100% for both genotypes. 80% reduction of blood flow was defined as sufficient MCAo (*n* = 10). Abbreviations. CCAo: Common Carotid Artery occlusion; MCAo: Middle Cerebral Artery occlusion; LDF: Laser Doppler flowmetry. (**b**) Infarct sizes of *Grina^−/−^* mice and wildtype littermates after 30 min of tMCAo followed by 6 and 72 h of reperfusion and EPO (5000 U/Kg) or saline (Vehicle) treatment were quantified (*n* = 6). (**c**) Modified neurologic severity score (mNSS) in a time-dependent manner (−1 h to 72 h) was assessed to grade neurological outcome after stroke. Sham (*n* = 8), wildtype littermates, (*n* = 10) *Grina^−/−^* (*n* = 10) mice. (**d**) Weight of all groups during the observation period and percent of losed weight at 6 and 72 h of reperfusion after stroke/sham surgery is shown in sham (*n* = 8), wildtype littermates (*n* = 10) and *Grina^−/−^* (*n* = 10) mice. Bars represent means ± SD. * *p* < 0.05 intergroup or treatment comparison, # *p* < 0.05 EPO treatment compared to the vehicle group.

Furthermore, no apparent differences in cerebral blood perfusion during MCA occlusion and at the beginning of reperfusion were found in both genotypes using intra-surgical laser doppler flowmetry (Figure 2a). The primary endpoint of the study was the evaluation of infarct sizes in TTC-stained brain sections of both genotypes after stroke followed by 6 or 72 h of reperfusion. A striatal lesion (mean ± SD, 13.42 mm^3^ ± 5.75 mm^3^) was found in wildtype mice after 6 h of reperfusion. The infarction showed a threefold progression at the end of the 72 h reperfusion period (41.63 mm^3^ ± 7.45 mm^3^). EPO administration substantially reduced the infarct sizes at both reperfusion time points in the wildtype mice. Here, we detected a reduction of nearly 80% (2.97 mm^3^ ± 2.01 mm^3^) after 6 h of reperfusion and a lowering of infarction by half after 72 h (19.47 mm^3^ ± 3.67 mm^3^). Strikingly, already after 6 h of reperfusion, *Grina^−/−^* mice revealed larger infarct sizes (50.77 ± 6.60 mm^3^) than wildtype mice after 72 h of reperfusion. In the progress of reperfusion, GRINA-deficient mice revealed an increase in lesion size of 68% after 72 h (85.47 ± 12.26mm^3^). In contrast to wildtype mice, EPO administration in *Grina^−/−^* mice did not result in a statistically significant reduction of the infarct sizes, neither at 6 h (37.73 ± 8.41 mm^3^) nor at 72 h of reperfusion (71.29 ± 12.01 mm^3^) (Figure 2b). Our findings indicate a crucial role for GRINA in both the development and progression of infarction. Of note, Rojas-Rivera et al. have suggested that GRINA might be a UPR target controlling cellular apoptosis [29], which could offer an explanation for the observed worsening of the disease process in GRINA-deficient mice.

### 2.2. Loss of GRINA Worsens Neurological Outcome And Abolishes EPO-mediated Neuroprotection after Ischemic Stroke

In order to examine the impact of stroke on behaviour and general neurological status, a modified neurologic severity score (mNSS) was assessed at five different time points (−1 h, 1 h, 6 h, 24 h, and 72 h after stroke) as previously described (Habib et al. 2019). Mild neurological deficits (0-4 points) were detected in mice directly after sham surgery which were no longer apparent after 6 h of reperfusion. Wildtype mice revealed moderate injuries (6.3 ± 1.27) in the early hours after stroke. Especially a flexion of forelimb and a weaker cage grasp when raising the mice by the tail were observed. In addition, besides cycling towards the paretic side, a missing whisker response to light touch, visual placing and missing pinna and corneal reflex were detected in wildtype mice in the first 6 h after stroke. In progress of reperfusion time, neurological deficits such as cycling recovered. GRINA-deficient mice revealed higher neurological impairments (8.1 ± 0.83) compared to WT mice after stroke. Interestingly, an EPO-dependent improvement of neurological outcome over the observation period was seen in WT mice but not in GRINA-deficient mice (Figure 2c). Furthermore, *Grina^-/-^* mice showed greater weight loss than WT mice at both reperfusion times (Figure 2d).

### 2.3. EPO Treatment Abrogated Stroke-induced Apoptosis in Both Genotypes after 6 and 72 h of Reperfusion

**Figure 3:**
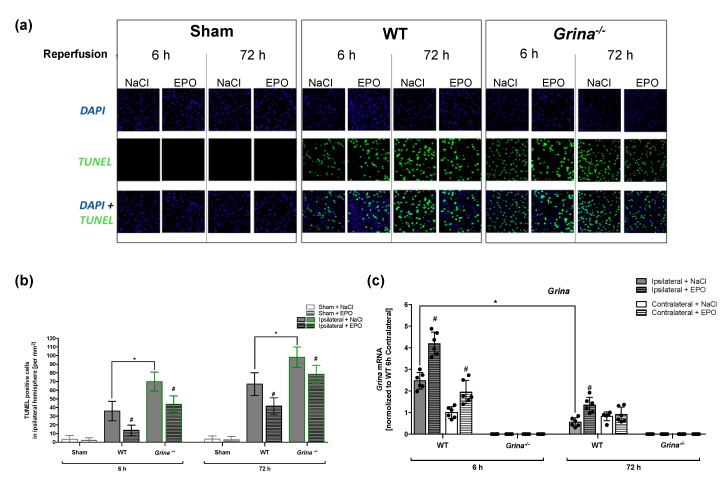
EPO administration abrogates stroke induced apoptosis after both 6 and 72 h of reperfusion (**a**) Representative images of TUNEL positive cells in paraffin-embedded coronal ipsilateral brain sections of mice sacrificed 6 and 72 h after 30 min of tMCAo or sham surgery are shown. Green fluorescence shows TUNEL-positive cells and blue fluorescence indicates DAPI staining of the nuclei. (**b**) Quantification of TUNEL-positive cells in all genotypes and treatment groups after 6 and 72 h of reperfusion is shown. Six ipsilateral brain slices per mouse (Bregma 0 ± 1 mm) with 12 × 0.01 mm^2^ grids per slice were counted by two individual investigators in a blinded manner (*n* = 4 animals). (**c**) *Grina* mRNA levels in the peri-infarct zone and the corresponding contralateral hemisphere after 30 min of tMCAo followed by 6 and 72 h of reperfusion are shown (*n* = 6 animals). Bars represent means ± SD. * *p* < 0.05 intergroup comparison, # *p* < 0.05 EPO treatment compared to the vehicle group.

We examined the extent of apoptosis after 30 min of tMCAo followed by 6 h or 72 h of reperfusion using TUNEL assay as previously described [33]. While sham-operated mice of both genotypes displayed nearly no apparent TUNEL positive/apoptotic cells, tMCAo significantly increased the number of apoptotic cells (sham vs. WT, 3.33 ± 4.30 vs. 34.7 ± 12.15 per mm^2^) (Figure 3a).

GRINA-deficiency (68.04 ± 13.47 per mm^2^) doubled the number of apoptotic cells 6 h after tMCAo compared to wildtype mice (34.7 ± 12.15 per mm^2^) (Figure 3b). In the progress of reperfusion, the number of TUNEL-positive cells increased in both genotypes. 72 h after stroke, *Grina^−/−^* mice (97.25 ± 12.94 per mm^2^) revealed 64% more apoptotic cells than wildtype mice (62.91 ± 17.60). Interestingly, in contrast to our volumetric evaluation of infarct sizes, EPO mitigated apoptosis rates not only in WT but also in GRINA-deficient mice at both reperfusion times after tMCAo. We detected 59% lower rates of apoptotic cells after 6 h and 29% after 72 h of reperfusion in wildtype mice. Similar tendencies were seen in *Grina^-/-^* mice with a decrease of 25% of TUNEL-positive cells after 6 h and of 15% after 72 h of reperfusion. Of note, administration of EPO induced the largest reduction of apoptosis in the early reperfusion period in both genotypes. To investigate the influence of both reperfusion times on *Grina* mRNA levels after ischemic stroke, we measured *Grina* mRNA levels in the peri-infarct zones and the corresponding contralateral hemispheres of both genotypes using RT-qPCR (Figure 3c). As expected, no *Grina* mRNA was detected in *Grina^−/−^* mice. In the WT mice, however, a 2.5-fold upregulation of *Grina* mRNA levels was observed in the peri-infarct zone compared to the contralateral hemisphere. EPO was able to significantly increase the mRNA level of *Grina* in both hemispheres. Interestingly, after a reperfusion time of 72 h *Grina* mRNA levels in the ipsilateral hemisphere were reduced by 50% compared to the contralateral hemisphere. Again, EPO administration was able to significantly upregulate *Grina* mRNA levels in the ipsilateral hemisphere. These data suggest that EPO-mediated reduction of apoptosis depends not only on the presence of *Grina*, but also suggests that other endogenous mechanisms might be involved in the neuroprotection conveyed by EPO.

### 2.4. EPO Enhances the Activation of the Pro-survival IRE1α Branch And Counteracts the Pro-apoptotic PERK Branch of UPR in the Early Phase after Ischemia/Reperfusion Injury

To study the impact of GRINA deficiency and EPO administration on the activation of the IRE1α and PERK branches of the UPR in a time-dependent manner after ischemic stroke, we used biopsies from the peri-infarct zones and the corresponding contralateral hemisphere of both genotypes after 6 and 72 h of reperfusion for gene expression analyses. To determine the activation of the most conserved IRE1α-XBP1 pathway after stroke, we measured spliced *Xbp1* mRNA (*Xbp1s*) and mRNA levels of its downstream target gene *Dnajb9* using RT-qPCR and performed an *Xbp1s* splicing detection assay.

**Figure 4:**
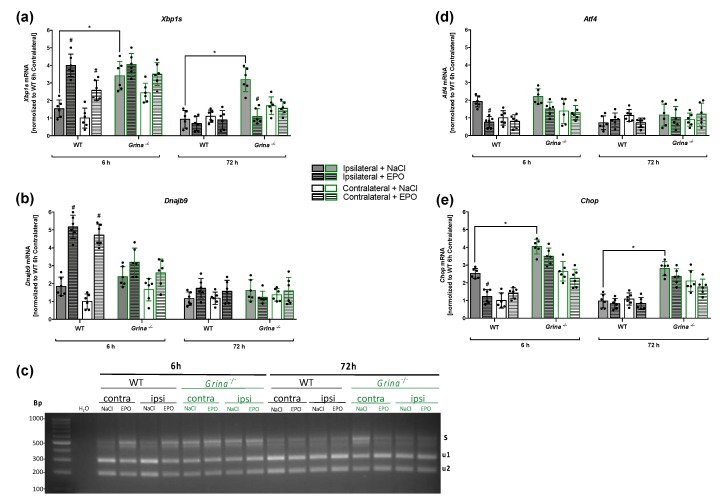
EPO enhances the activation of pro-survival IRE1α and counteracts the pro-apoptotic PERK-branch of UPR in the early phase after ischemia/reperfusion injuryBiopsis of the peri-infarct zone and the coresponding contralateral hemiphere from EPO or saline treated *Grina^−/−^* mice and wildtype littermates after 30 min of tMCAo followed by 6 and 72 h of reperfusion were used for gene expression analyses (*n* = 6). To evaluate the activation of the IRE1α after stroke, mRNA levels of *Xbp1s* (**a**) and *Dnajb9* (**b**) were measured using RT-qPCR. (**c**) The expression of spliced *Xbp1s* mRNA was evaluated by an *Xbp1* splicing detection assay. cDNA was used to amplify *Xbp1* by PCR. *Xbp1*u amplicon digestion by PstI resulted in two fragments (u1, u2). Size of *Xbp1s* amplicon lacking PstI sites was 473bp. To monitor PERK activation, mRNA levels of *Atf4* (**d**) and *Chop* (**e**) were measured using RT-qPCR. Bars represent means ± SD. *n* = 6 of all genotypes and treatment groups. * *p* < 0.05 intergroup comparison, # *p* < 0.05 EPO treatment compared to the vehicle group.

Stroke increased *Xbp1s* mRNA levels, however GRINA-deficiency revealed significantly higher *Xbp1s* mRNA levels than WT mice 6 h after stroke (*p* = 0.0043) (Figure 4a).

The same tendencies were detected 72 h after stroke with *Grina^−/−^* mice displaying significant higher *Xbp1s* mRNA levels compared to wildtype mice (*p* = 0.0041). While *Xbp1s* mRNA levels in *Grina^−/−^* mice remained at same levels at both reperfusion times, the levels of *Xbp1s* mRNA decreased in wildtype mice 72 h after tMCAo. EPO administration further enhanced the *Xbp1s* mRNA levels, but a statistically significant elevation of mRNA levels was only found in wildtype mice (*p* = 0.0021) 6 h after stroke. Noteworthy, the injection of EPO significantly reduced the elevated *Xbp1s* mRNA levels in GRINA-deficient mice after 72 h of reperfusion (*p* = 0.0053) (Figure 4a). The spliced form of *Xbp1* mRNA encodes the XBP1s protein, which as a transcription factor induces the expression of *Dnajb9*. Similar to *Xbp1s* mRNA levels, stroke promoted stronger *Dnajb9* mRNA expression in both genotypes after 6 h of reperfusion. EPO administration further boosted *Dnajb9* mRNA levels in wildtype mice after 6 h of reperfusion (*p* = 0.0006) (Figure 4b). However, the mRNA levels of *Dnajb9* were overall reduced and showed no significant differences within the genotypes 72 h after tMCAo. Activation of the IRE1α RNase domain is known to mediate splicing of the *Xbp1* mRNA by removing a 26-nucleotide intron leading to a shift in the codon reading frame. The spliced *Xbp1* mRNA is translated into a functional and stable transcription factor of the UPR. To visualize *Xbp1s* PCR products after ischemic stroke and upon EPO treatment, we performed an *Xbp1s* splicing detection assay (Figure 4c). In line with our RT-qPCR data of *Xbp1s* mRNA (Figure 4a), strongest generation of *Xbp1s* was detected after the early reperfusion time (6 h) and was boosted after EPO application. In order to monitor the activation of the PERK arm of the UPR, we examined the mRNA levels of *Atf4* and its downstream gene *Chop*, which is attributed to promote apoptosis. The mRNA levels of *Atf4* were slightly increased in wildtype and *Grina^−/−^* mice 6 h after stroke. The later reperfusion time showed no apparent differences of *Atf4* mRNA levels between both genotypes. EPO administration, however, significantly counteracted this effect only in wildtype mice (*p* = 0.0102) (Figure 4d). Stroke promoted an increase of *Chop* mRNA in wildtype mice after 6 h of reperfusion and EPO significantly mitigated this effect (*p* = 0.0355) (Figure 4e). GRINA-deficiency, however, revealed significantly higher *Chop* mRNA levels at both 6 h (*p* = 0.0007) and 72 h (*p* = 0.0001) after stroke compared to wildtype mice. Here, a tendency towards a reduction of *Chop* mRNA was indicated after EPO administration, but this did not reach statistical significance.

Our findings demonstrate stroke-induced activation of the IRE1α and PERK pathway of the UPR, especially in the early (6 h) reperfusion phase. This suggests that ER stress and the associated activation of the UPR might play an important role in the initial phase after stroke.

### 2.5. GRINA-deficiency Leads to Increased Apoptosis And Activation of the IRE1α And PERK Arm of UPR in Primary Murine Cortical Cells after OGD

**Figure 5:**
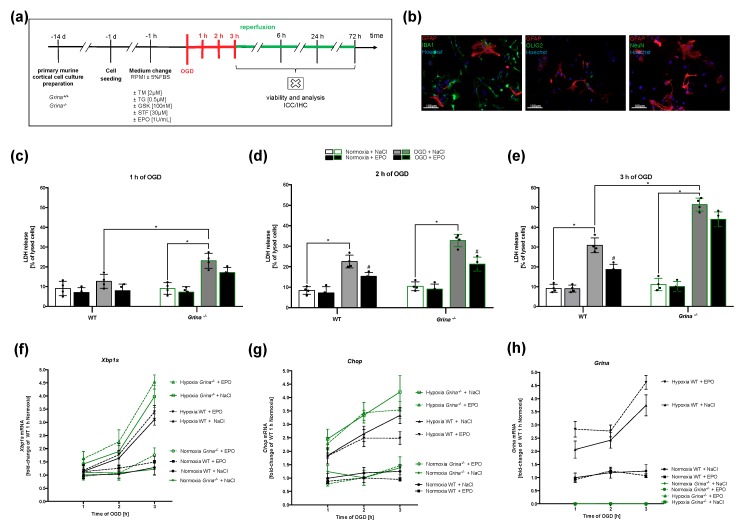
OGD for 3 h induced high rates of apoptosis and activated the IRE1α and PERK arm of UPR (**a**) Experimental design of OGD with primary murine cortical mixed cell culture. OGD was performed for either 1, 2 or 3 h followed by a reperfusion of 1, 6, 24 and 72 h. Prior to OGD/normoxia (−1 h), administration of TM (2 µg/mL), TG (0.5 µM), GSK (100 nM), STF (30 µM) and treatment with EPO [1 U/mL] or corresponding controls (NaCl/DMSO) was performed. (**b**) Representative images of immunofluorescence staining using anti-GFAP antibody to stain astrocytes (red), anti-IBA1 for microglia staining (green), anti-OLIG2 for oligodendrocyte staining (green) and anti-NeuN antibody for staining neurons (green). DNA staining was performed using Hoechst (blue). Primary murine cortical mixed cell culture contained astroytes, microglia and oligodendrocytes but no neurons. LDH release in *Grina^−/−^* and wildtype littermate primary murine cortical cells was determined after 1 h (**c**), 2 h (**d**), 3 h (**e**). The mRNA levels of *Xbp1s* (**f**), *Chop* (**g**) and *Grina* (**h**) after 1, 2 and 3 h of OGD are shown. Bars represent means ± SD. *n* = 4 individual cell culture preperations from mice pups (P0-P2) of all genotypes and treatment groups. * *p* < 0.05 intergroup comparison, # *p* < 0.05 EPO treatment compared to the vehicle group.

To support our in vivo findings and to further mechanistically substantiate the described results, we subjected primary murine cortical cells from both genotypes to OGD of different duration (1–3 h) (Figure 5a). We were interested in examining the OGD tolerance of both genotypes upon EPO treatment. In addition, we intended to investigate the impact of EPO on the activation levels of the IRE1α and PERK arms of the UPR after OGD.

First, we introduced the primary murine cortical cells consisting of GFAP-positive astrocytes, OLIG2-positive oligodendrocytes, and IBA1-positive microglial cells (Figure 5b) to either 1 h, 2 h or 3 h of OGD and measured cell death using an LDH release assay. While there was no significant increase in cell death in wildtype cells (12%), an almost doubled cell death rate was detected in GRINA-deficient cells (23.3%) after 1 h of OGD. EPO administration had no beneficial effects in both genotypes after 1 h of OGD (Figure 5c). However, prolonging the OGD duration to 2 h promoted significant higher cell death rates in both genotypes. Interestingly, EPO significantly reduced cell death in both genotypes after 2 h of OGD (WT: from 24% to 14%, *Grina^−/−^*: from 33% to 21%) (Figure 5d). The EPO-dependent reduction of the cell death rate was also observed after 3 h of OGD in wildtype cells (from 32% to 18%). However, high cell death rates in GRINA-deficient cells after 3 h of OGD (48%) were not be significantly reduced by EPO (39%) (Figure 5e). Increasing OGD duration was accompanied by a stronger activation of the IRE1α and PERK branch of the UPR. Only after 3 h of OGD a significant increase of *Xbp1s* mRNA levels was observed in both genotypes (Figure 5f). Comparable to our in vivo results, an EPO-dependent increase of *Xbp1s* mRNA levels was observed at each time point of OGD treatment. In contrast to *Xbp1s* mRNA expression, a significant increase of *Chop* mRNA levels was observed in both genotypes already after an OGD duration of 2 h. Also here, the strongest increase of *Chop* mRNA was observed after 3 h OGD, with GRINA-deficient cells always showing higher *Chop* mRNA levels than wildtype cells. EPO administration significantly reduced *Chop* mRNA levels only in wildtype cells (Figure 5g). We also addressed the *Grina* mRNA expression after the three analyzed mentioned OGD times to investigate the temporal regulatory process. OGD increased the mRNA levels of *Grina*, which was further enhanced by EPO administration (Figure 5h).

### 2.6. OGD Activates the IRE1α And PERK Arms of the UPR with a Similar Tendency as Pharmacological Treatment with Tunicamycin (TM) and Thapsigargin (TG)

Next, we compared the extent of OGD-dependent activation of the IRE1α and PERK arms of the UPR upon pharmacological induction of the UPR by means of TM and TG treatment. Thus, primary murine cortical cells of both genotypes were treated with TM (2 µg/mL) or TG (0.5 µM) for 4 h or subjected to normoxia/OGD for 3 h (Figure 5a).

For the activation of the IRE1α branch of UPR after stroke, mRNA levels of *Xbp1s* and *Dnajb9* were measured in GRINA-deficient and wildtype primary murine cortical cells using RT-qPCR. In addition, we performed an *Xbp1* splicing detection assay to visualize spliced *Xbp1s* mRNA.

**Figure 6:**
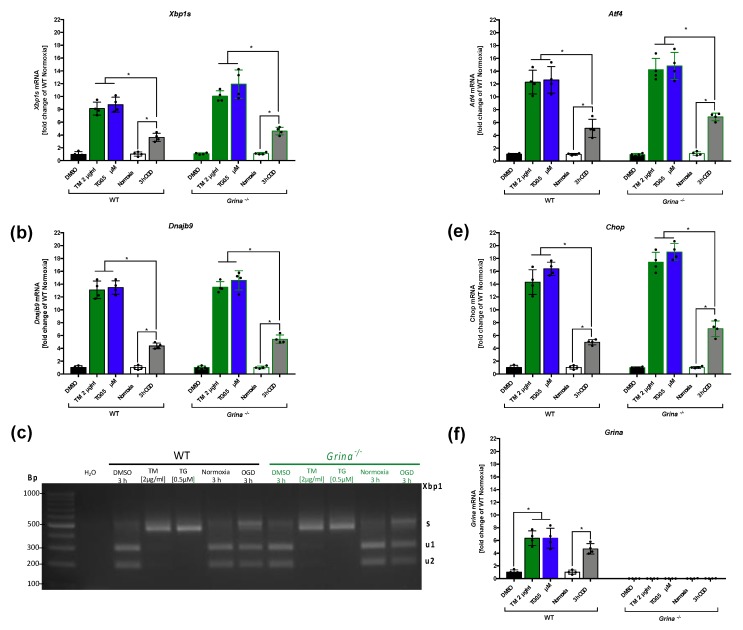
OGD activated the IRE1α and PERK arm of the UPR with similar tendency as pharmacological treatment with Tunicamycin and ThapsigarginPrimary murine cortical cells were treated with TM (2 µg/mL) or TG (0.5 µM) for 4 h or subjected to OGD/normoxia for 3 h. For the activation of the IRE1α branch of UPR after stroke, mRNA levels of *Xbp1s* (**a**) and *Dnajb9* (**b**) were measured in GRINA-deficient and wildtype primary murine cortical cells using RT-qPCR. The expression of spliced *Xbp1s* mRNA was evaluated by an *Xbp1* splicing detection assay (**c**). To evaluate PERK activation, mRNA levels of *Atf4* (**d**) and *Chop* (**e**) were measured using RT-qPCR. The impact of TM, TG and 3 h of OGD on *Grina* mRNA levels in GRINA-deficient and wildtype primary murine cortical cells using RT-qPCR is shown (**f**). Bars represent means ± SD. *n* = 4 individual cell culture preperations from mice pups (P0–P2) of all genotypes and treatment groups. * *p* < 0.05 intergroup comparison.

OGD induced significantly higher levels of *Xbp1s* mRNA in both genotypes compared to normoxia control (WT: *p* = 0.0051, *Grina^−/−^*: *p* = 0.0012). Stimulation with TM (vs. WT: *p* = 0.032, vs. *Grina^−/−^*: *p* = 0.035) and TG (vs. WT *p* = 0.014, vs. *Grina^−/−^* *p* = 0.0077) showed significantly enhanced *Xbp1s* mRNA levels compared to OGD in both genotypes (Figure 6a).

Comparable tendencies were observed for *Dnajb9* mRNA levels (Figure 6b). In line with RT-qPCR data for *Xbp1s* (Figure 6a), spliced *Xbp1s* mRNA levels were highly abundant after OGD, and strongly elevated after TM and TG treatments in both genotypes (Figure 6c). To evaluate PERK activation, mRNA levels of *Atf4* and *Chop* were measured using RT-qPCR (Figure 6d,e). Here, OGD induced a four-fold increase of *Atf4* mRNA levels in wildtype cells and a five-fold increase in *Grina^−/−^* cells compared to corresponding normoxic conditions. TM and TG treatment yielded a 12–14-fold increase of *Atf4* mRNA in both genotypes compared to normoxia and DMSO control (Figure 6d). For mRNA levels of *Chop*, we observed a four-fold elevation in wildtype and a seven-fold increase in GRINA-deficient cells after OGD compared to normoxic condtions (Figure 6e). A further 3–4-fold increase of *Chop* mRNA was detected after TM and TG stimulation in both genotypes compared to the OGD group.

### 2.7. Both EPO and PERK-inhibitor GSK-2606414 Reduced Cell Death And Regulated Grina mRNA Levels after OGD/Reoxygenation

**Figure 7:**
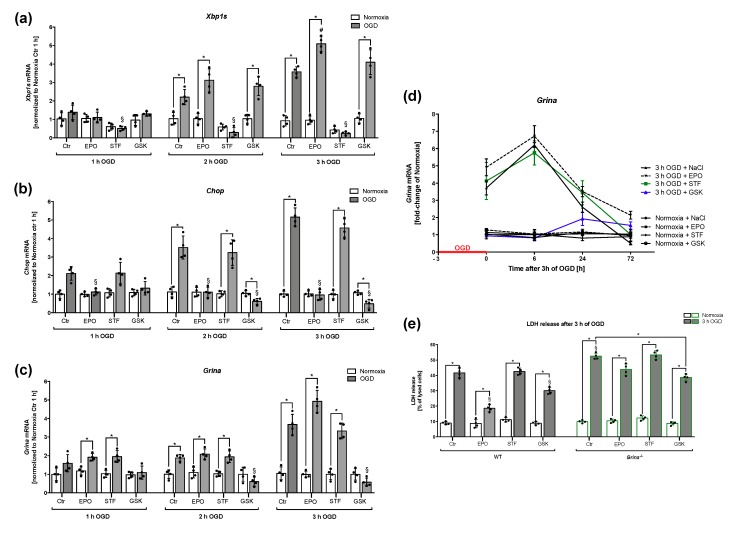
EPO administration and inhibition of the PERK arm of UPR reduced cell deatand regulated *Grina* mRNA levels after OGD/reoxyginationTreatment with PERK-inhibitor GSK [100 nM], IRE1α-inhibitor STF (30 µM), EPO (1 U/mL) or corresponding controls (NaCl/DMSO) was performed 1 h prior to either 1 h, 2 h or 3 h of OGD in wildtype murine cortical cells. The mRNA levels of *Xbp1s* (**a**), *Chop* (**b**) and *Grina* (**c**) after 1 h, 2 and 3 h of OGD are shown. (**d**) The impact of STF, GSK and EPO on *Grina* mRNA levels in wildtype primary murine cortical cells after 3 h of OGD followed by 6 h, 24 and 72 h of reperfusion is shown. (**e**) LDH release in *Grina^−/−^* and wildtype primary murine cortical cells was determined after 3 h of OGD. Bars represent means ± SD. *n* = 4 individual cell culture preperations from mice pups (P0–P2) of all genotypes and treatment groups. * *p* < 0.05 intergroup or treatment comparison, # *p* < 0.05 EPO treatment compared to the vehicle group, § *p* < 0.05 compared to wildtype control (Ctr).

Finally, we aimed to elucidate the impact of IRE1α and PERK inhibition on cell death and *Grina* expression and to compare these with EPO treatment after OGD. Thus, we treated wildtype murine cortical cells with the PERK-inhibitor GSK-2606414 (GSK; 100 nM), the IRE1α-inhibitor STF-083010 (STF; 30 µM), EPO (1 U/mL) or corresponding controls (NaCl/DMSO) 1 h prior to either 1, 2 or 3 h of OGD. We evaluated mRNA levels of *Xbp1s*, *Chop*, *Grina* and the release of LDH after 1, 2 and 3 h of OGD. Comparable with our previous experiments, the mRNA levels of *Xbp1s* were found elevated with increasing OGD duration, displaying highest levels after 3 h of OGD (Figure 7a).

EPO further enhanced mRNA levels of *Xbp1s*. As expected, treatment with STF for 4 h significantly decreased *Xbp1s* mRNA compared to the corresponding control after OGD (2 h OGD: *p* = 0.0051, 3 h OGD: *p* < 0.0001). Interestingly, STF treatment also reduced the mRNA level of *Xbp1s* under normoxic conditions, indicating basal IRE1α RNase activity in primary murine cortical cells. STF administration had no effect on *Chop* mRNA levels (Figure 7a). Both GSK and EPO treatment were able to significantly attenuate OGD-dependent upregulation of *Chop* mRNA after 2 and 3 h of OGD. GSK application even managed to reduce the level of *Chop* mRNA under the level of normoxic conditions after 2 h (*p* = 0.0305) and 3 h (*p* = 0.0156) of OGD. Consistent with our previous experiments on *Grina* regulation after different OGD durations, *Grina* expression was upregulated after OGD and further enhanced by EPO (Figure 7c). However, while STF seemed to have no effect on *Grina* mRNA regulation after OGD, GSK clearly down-regulated *Grina* mRNA levels after 2 and 3 h of OGD compared to control (2 h: *p* = 0.0034, 3 h: *p* = 0.0023). Next, we investigated the influence of the above mentioned inhibitors and EPO administration on *Grina* mRNA expression after 3 h of OGD with subsequent reperfusion times of up to 72 h. OGD substantially increased *Grina* mRNA levels, which was further enhanced by EPO compared to normoxic conditions. Over the course of reperfusion, both EPO and the vehicle group showed a further increase after 6 h, but then decreased significantly over time. Only the EPO-treated cells showed a significantly higher *Grina* expression after 72 h compared to the vehicle control. While STF had no obvious effect on *Grina* expression, GSK reduced it significantly after OGD. This reduction lasted for 6 h and afterwards showed an increase of *Grina* mRNA up to 72 h. This result indicates that *Grina* after OGD is regulated by the PERK arm of the UPR. We then investigated the influence of both inhibitors and EPO on cell viability after 3 h of OGD using an LDH release assay. A marked OGD-dependent increase of LDH in wildtype cells was observed compared to the control. In GRINA-deficient cells a further increase of 28.3% was evident after OGD compared to wildtype cells. After EPO treatment the cell death rate decreased by 54.5% in wildtype cells and by 18.5% in GRINA-deficient cells. Interestingly, GSK managed to reduce the cell death rate in both genotypes in a similar way (WT: 27.4%, *Grina^−/−^*: 25.9%). Although GSK seemed to regulate *Grina* expression, there was no significant difference in cell viability after OGD in both genotypes. These data suggest that reduction of *Chop* mRNA by both GSK and EPO might lead to cytoprotection.

## 3. Discussion

In the present study, we demonstrated that transient ischemic stroke in mice and OGD in primary murine cortical glial cell cultures activated the IRE1α and PERK arm of the UPR. This activation was particularly observed in the initial phase (6 h) after ischemia (tMCAo/OGD) and abated during the reperfusion time of 72 h. EPO administration promoted the activation of the adaptive, pro-survival IRE1α branch of the UPR both in vitro and in vivo and counteracted the terminal, pro-apoptotic PERK pathway of the UPR. Furthermore, we pointed out that the PERK inhibitor GSK-2606414, but not IRE1α inhibitor STF-083010, reduced the cell death rate and altered *Grina* transcription. GRINA-deficiency not only promoted larger infarct volumes, larger neurological deficits and more apoptosis after both 6 and 72 h of reperfusion, but also showed increased activation of both above mentioned UPR branches compared to wild type animals.

Based on our previous results that GRINA deficiency 72 h after a 30 min MCAo leads to larger infarct sizes and clinical deficits [33], we here investigated the influence of GRINA on the infarct progression. GRINA-deficient mice showed larger infarct volumes, worse outcomes and a higher number of apoptotic cells than WT mice both after 6 h and after 72 h of reperfusion. In fact, lesion sizes in *Grina^−/−^* mice were larger after 6 h than in wildtype mice after 72 h of reperfusion. This implies that GRINA might be directly involved in post-ischemic apoptosis. Due to the fact that the cerebral damage and associated neurological deficits in wildtype mice always remained below the level of GRINA-deficient mice over the observation period of 72 h, GRINA also might play a crucial role in neuro-restoration. Here it would be interesting to investigate whether a longer duration of ischemia (1 h) followed by longer periods of reperfusion-time (e.g., 7 d, 14 d) supports these results, or whether the damage in wildtype mice might converge to that of GRINA-deficient mice over time.

In line with our previous results, *Grina* mRNA levels were reduced in the peri-infarct zone after a reperfusion time of 72 h and EPO was able to increase *Grina* mRNA levels. Interestingly, *Grina* mRNA levels were elevated in the initial phase after ischemia/OGD (6 h) and showed a reduction during the 72 h reperfusion period. Noteworthy, EPO treatment led to an increase in *Grina* expression at any time of reperfusion. The absence of EPO-dependent neuroprotection in the GRINA-deficient mice over the entire observation period of 72 h suggests an important role of GRINA in EPO-promoted pro-survival mechanisms. However, it should be noted that in the early phase after stroke (6 h) EPO treatment reduced apoptosis rates in GRINA-deficient mice too. This is likely attributed to the pleiotropic effect of EPO, which seems to be partly independent of GRINA and mediated by the regulation of the post-ischemic UPR. Especially in the early phase after ischemia/OGD, a strong activation of the two most conserved UPR pathways, IRE1α and PERK, was evident. EPO administration boosted the activation of the IRE1α arm and down-regulated the PERK branch of the UPR. Chiang and colleagues identified a novel ATF4 binding site (TGACCTCT) within the EPO 3’-enhancer region using chromatin immunoprecipitation analysis and have shown that the induction of ER stress in rat liver and kidney by Tunicamycin decreased the hepatic and renal mRNA and plasma level of EPO [46]. However, to our knowledge, the influence of exogenous EPO on ER stress and UPR and especially the involvement of EPO in the UPR after ischemic stroke has not been investigated yet.

It is well established that ischemic stroke impairs ER function and activates the UPR [47], but less is known about the mechanisms that link the UPR and downstream cascades to stroke outcome. The UPR is divided into two phases: the adaptive phase of the UPR is responsible for restoring ER homeostasis during low or transient ER stress and the terminal phase of the UPR promotes apoptotic processes upon high and/or sustained ER stress levels [48,49]. It is widely accepted that GRP78/BIP is bound to three stress sensors ATF6, IRE1α, and PERK in non-stressed cells, thereby keeping them in a resting state. Upon accumulation of unfolded proteins in the ER, GRP78/BIP dissociates from the stress sensors resulting in their activation to promote the protein folding capacity of the ER [50]. Among the three UPR sensors, the PERK branch is believed to be activated first in response to ER stress, followed by IREα and ATF6. The latter, however, seems to be not or only slightly activated after both transient global and focal cerebral ischemia [28,51]. We therefore focused on the IRE1α and the PERK arm of the UPR and compared a pharmacological activation of the UPR by Tunicamycin, an inhibitor of ER protein glycosylation, or Thapsigargin, an irreversible inhibitor of the ER calcium pump SERCA, with OGD. After 3 h of OGD both UPR arms showed a similar tendency in activation as after pharmacological treatment. Based on our in vivo data wherein we observed activation of the IRE1α and PERK arms especially in the early phase (6 h) after stroke, we assumed that activation of UPR is an early endogenous pro-survival response after stroke. In our in vitro experiments we found the strongest activation of the IRE1α and PERK arm after 3 h of OGD. The inhibition of the PERK and IRE1α arm of the UPR by GSK-2606414 and STF-083010, respectively, showed a sufficient down-regulation of the target genes Chop and Xbp1s after 1, 2 and 3 h of OGD. While STF-083010 treatment had no effect on *Grina* expression, the inhibition of the PERK by GSK-2606414 significantly decreased the *Grina* mRNA levels. The downregulation of *Grina* mRNA upon GSK-2606414 treatment continued up to 24 h after OGD and then gradually increased again. Rojas-Rivera et al. proposed that *Grina* expression might be a downstream effector of the PERK and ATF4 signaling branch that regulates cell survival under ER stress conditions, since they observed a significant reduction of *Grina* mRNA levels in *Atf4^−/−^* mice after Tunicamycin injection compared with WT control animals [29]. In accordance, our results suggest that a genetic or pharmacological inhibition of the PERK/ATF4 arm down-regulates the expression of *Grina*. The inhibition of the PERK arm of the UPR by GSK-2606414 does not seem to be as specific as assumed. It turned out that GSK-2606414 is not only a PERK inhibitor but also a potent RIPK1 inhibitor. The cytoprotective effects of GSK-2606414 we observed after OGD could be explained by the suppression of CHOP- and RIPK1-dependent apoptosis or necroptosis [52]. Moreno and colleagues recently proposed that GSK-2606414 penetrates the blood-brain barrier and abrogated the development of clinical prion disease in mice, with neuroprotection observed throughout the mouse brain [53]. While the use of EPO as single-agent therapy after ischemic stroke appears to be safe, the innocuousness of the PERK inhibitor GSK-2606414 has yet to be evaluated pre-clinically. Overall, the inhibition of PERK seems to offer a promising therapy option after stroke, hence further research on GSK-2606414 and/or other PERK inhibitors is very reasonable.

## 4. Materials and Methods

All experimental procedures described in this manuscript were approved by the District Government of North Rhine Westphalia in Recklinghausen, Germany (LANUV ID 84-20.04.2015.A292, approved on 20 April 2015) and experiments involving animals comply with the ARRIVE guidelines from the NC3R^s^ (National Centre for the Replacement, Refinement & Reduction of animals in research).

### 4.1. Mice

Embryos of *Grina^−/−^* mice were provided by the public Mutant Murine Regional Resource Centers (RRID: MMRRC_031871-UNC) and were generated by targeting the initiator codon situated on exon 2 of the *Grina* allele with a loxP-flanked neomycin cassette as previously described by Nielsen et al., 2011 and Habib et al. 2019. The B6.129(FVB)-*Grina^tm1.1Ldh^* background had been backcrossed to C57BL/6J (RRID:IMSR_JAX:000664) for more than ten generations. *Grina^−/−^* mice and their wildtype littermates were bred by the Institut für Versuchstierkunde, Universitätsklinik Aachen, RWTH Aachen University and were housed and handled in accordance with the guidelines of the Federation for European Laboratory Animal Science Associations (FELASA) in a pathogen-free, temperature-controlled (20–24 °C) facility with a 12/12 h light/dark cycle and access to pelleted food and water ad libitum.

### 4.2. Transient Middle Cerebral Artery Occlusion (tMCAo) And Study Protocol

For this randomized and blinded controlled trial a total of 120 male *Grina^−/−^* and wildtype littermate mice (10–12 weeks of age and 24–30 g weight) were assigned (Figure 1a). The study design is shown in the time-line diagram in Figure 1b. To abolish protective effects of gonadal steroid hormones as previously described [54,55], we only included male mice. tMCAo was performed for 30 min followed by 6 h or 72 h of reperfusion as previously described [33]. In brief, we induced anesthesia with 3% of isoflurane in 30% O_2_ balanced with N_2_O and maintained anesthesia in 1% isoflurane in 30% O_2_ and 69% N_2_O during surgery. The regional cerebral blood flow (rCBF) was measured with a laser doppler probe (Moor Instruments VMS-LDF2, Axminster, UK) affixed to the skull above the left MCA territory after the overlaying temporal bone was exposed by an incision. In supine position through a midline neck incision (<1 cm) the left common carotid artery (CCA) and the external carotid artery (ECA) were isolated and ligated. A 0.19 mm thick silicon coated filament (Doccol, #602212PK10Re) was threaded into the internal carotid artery (ICA) for MCA occlusion. A sufficient occlusion was confirmed by reduction in rCBF to <20% of the baseline (Figure 2a). Body temperature during surgery was maintained at 37 °C ± 0.5 °C using a feedback-controlled heating pad and a heating lamp. After 30 min of tMCAo, mice were supplemented with 0.5 mL saline i.p. and placed into a temperature-controlled incubator before returning to their home cages for the post-surgical survival period of 72 h. Body weight and temperature were measured daily. For pain relief, the animals were given s.c. buprenorphine (0.5 mg/kg body weight) directly after tMCAo/sham surgery and every 8 h over the entire observation period of 3 days. The sham group (32 mice) received the same surgical procedure, except from the filament insertion. A technical assistant who was not involved in the analysis identified the mice by earmark numbers and randomly assigned the mice to the experimental groups (Figure 1a). The surgeon had to demonstrate his ability to perform the MCAo procedure with a high degree of reproducibility prior to the study in order to reduce animal number and suffering and was blinded for genotype. Clinical examinations, data acquisition and data analysis were performed by investigators who were blinded to genotype and group assignment. The primary endpoint of this study was the infarct size after 6 and 72 h of reperfusion. Decapitation of mice was performed after isoflurane overdose. All surgical procedures were performed from 8.00 am to 11:30 am.

### 4.3. EPO Application And Exclusion Criteria

Recombinant human EPO (rhEPO) (Epoetin alfa Hexal, Hexal, Holzkirchen, Germany) was diluted in 0.9% NaCl and EPO doses of 5,000 U/kg body weight were given intra-peritoneally directly after 30 min of tMCAo, 24 and 48 h after reperfusion as previously described [33]. Controls were injected with saline only (vehicle). Mice would be excluded from further analysis if a reduction of rCBF >80% of baseline and a recovery of rCBF to CCAo levels (60–70% of baseline) after 5–10 min reperfusion (Figure 2a) was missing. Furthermore, animals with brain hemorrhage, seizures, extensive weight loss (>20% of baseline), missing infarction in TTC-staining, and those that did not develop sufficient neurological deficits (mNSS < 5) were excluded. Mice that died during the observation period of 72 h were excluded from all analyses, except mortality rate between genotypes and treatment (Figure 1a).

### 4.4. Modified Neurologic Severity Score (mNSS)

For the evaluation of clinical outcome and focal neurological dysfunction after tMCAo, we assessed a modified neurologic severity score (mNSS) as previously described [33]. Our test battery separately graded motor function (body asymmetry, muscle status, abnormal movement and gait), sensory function (visual, tactile and proprioceptive) and reflexes (corneal reflex, pinna reflex, whisker response to light touch, startle reflex). The score ranges from 0 (no deficits) to 15 points representing the poorest performance in all items and is calculated as the sum of the general and focal deficits. For the inability to perform the task, abnormal performance or for lack of a tested reflex one point was awarded. We defined a score of 1–5 as mild, 6–10 as moderate, and 11–15 as severe neurological deficits. Two individual investigators, other than the experimenter and blinded to genotype and treatment of the mice, examined each animal 1 h before and 1, 6, 24, 48 and 72 h after tMCAo or sham surgery (Figure 1b).

### 4.5. Volumetric Evaluation of Infarct Sizes and Hematology

For the primary endpoint of our study, infarct volumes were measured using the 2,3,5-triphenyltetrazolium chloride staining method as described earlier [54]. In brief, 6 h or 72 h after surgery mice were deeply anesthetized and EDTA blood of each animal was taken transcardially for a blood count using the Celltac α MEK-6450K (Nihon Kohden Europe, Rosbach, Germany) in the Institut für Versuchstierkunde, Universitätsklinik Aachen, RWTH Aachen University (Table 1). The brains were removed immediately and sliced coronally. The 1-mm-thick brain sections were incubated in 2% TTC (Sigma Aldrich, St. Louis, MO, USA) for 10 min at 37 °C followed by a fixation in 10% formaldehyde in phosphate-buffered saline (PBS). The stained sections were photographed (Fujifilm X-T20, XF18-55mm) and evaluated in a blinded manner using ImageJ software (NIH, Bethesda, MD, USA). The infarct volume was corrected for brain edema with the following equation: Corrected ischemic lesion (CIL) = measured ischemic lesion (MIL) − (Ipsilateral hemisphere (IH) − contralateral hemisphere (CH)). Total infarct volumes were calculated by adding the mean area of each section and multiplied by the thickness of the sections.

### 4.6. Terminal Deoxynucleotidyl Transferase dUTP Nick End Labeling (TUNEL)

Apoptosis/necrosis after 30 min of tMCAo or sham surgery followed by 6 h or 72 h of reperfusion was measured by TUNEL assay using In Situ Cell Death Detection Kit (Roche, #11684795910, Basel, Switzerland) and DAPI (CAS-Nr. 28718-90-3, #6335, Roth, Karlsruhe, Germany) according to the manufacturer’s instructions as previously described [33]. In brief, paraffin-embedded 5 µm (Bregma 0 ± 1 mm) tissue sections were de-paraffinized, rehydrated, treated with proteinase K working solution, and permeabilized. Permeabilized tissue sections were incubated with the TUNEL reaction mixture in a humidified atmosphere for 1 h at 37 °C in the dark. Sections were counterstained for nuclei with DAPI for 20 s and coverslipped using fluorescent mounting medium (Dako, Agilent Technologies, Santa Clara, CA, USA), and observed under a fluorescence microscope (Leica DMI 6000 B, Leica Microsystems, 35578, Wetzlar, Germany). The results were quantified by counting the number of TUNEL-positive cells in 12 × 0.01 mm^2^ grids per ischemic brain sections obtained from four animals per group.

### 4.7. Immunocytochemistry

Immunocytochemistry was used in primary murine cortical cells of post-natal (P0–P2) mouse pups to identify and distinguish between astrocytes (anti-GFAP-antibody, sc6170, RRID: AB_641021, 1:300), oligodendrocytes (anti-Olig2, Millipore, 64293, Darmstadt, Germany), AB9610, RRID: AB_570666, 1:500), microglia (anti-Iba1-antibody, wako, RRID: AB_839504, 1:1000) and neurons (anti-NeuN-antibody, Millipore, MAB377, RRID:AB_177621, 1:500) as previously described [56,57]. In brief, 5 × 10^4^ cells were seeded on cover slips on a 24-well-plate. Fixation with 3.7% PFA in PBS was performed for 30 min at room temperature. Prior to staining Triton X-100 was added to the cells and the unspecific sites were blocked with IFF buffer for 1 h. Primary antibodies were diluted in IFF and incubated overnight at 4 °C. Secondary antibodies (1:500) diluted in IFF were added for 1 h at room temperature. After washing three times with PBS, the nuclei were stained with Hoechst (Invitrogen, 76131, Karlsruhe, Germany). Fluorescence pictures were taken with a Leica DMI 6000 B (Leica Microsystems, 35578, Wetzlar, Germany).

### 4.8. Reverse-Transcription Quantitative PCR (RT-qPCR)

Gene expression analyses were performed with tissue biopsies from the peri-infarct areas (Bregma 0 ± 1 mm) and the corresponding contralateral hemisphere using a stereomicroscopic approach or with glial cells after oxygen-glucose-deprivation. After dissolving and homogenizing tissue or cells in PeqGold (PeqLab #30-2010, Germany) total RNA was extracted using the peqGold RNA TriFast as previously described [55]. Complementary DNA was synthesized using the MMLV reverse transcription kit and random hexanucleotide primers (Invitrogen, Germany) using 1 μg of total RNA. Triplicates of every sample were transferred by a pipetting robot (Corbett CAS-1200) to Rotor-Gene strip reaction tubes (Starlab, 22143, Hamburg, Germany) and RT-qPCR analysis was performed using the Rotor-Gene Q device (Qiagen, 40724, Hilden, Germany). RNase free H_2_O (Merck, 64293, Darmstadt, Germany) served as no template control (NTC) and primer efficiencies were calculated using the Pfaffl method [58]. The target genes and two housekeeping genes, glucuronidase beta (*Gusb*) and glyceraldehyde-3-phosphate dehydrogenase (*Gapdh*) were measured at cycle threshold (Ct values) and relative quantification was calculated by the ΔΔCt method using the qbase+ software (Biogazelle, Belgium). Data are expressed as relative amount of the target genes to *Gusb* and *Gapdh* respectively. The following forward (fwd) and reverse (rev) primers were used (5’–>3’): *Atf4* (fwd: CCT GAA CAG CGA AGT GTT GG; rev: TGG AGA ACC CAT GAG GTT TCA A), *Chop* (fwd: ACG GAA ACA GAG TGG TCA GTG C; rev: CAG GAG GTG ATG CCC ACT GTT C), *Dnajb9* (fwd: CCC CAG TGT CAA ACT GTA CCA G; rev: AGC GTT TCC AAT TTT CCA TAA ATT), *Gapdh* (fwd: ACT CAA GAT TGT CAG CAA TGC A; rev: TGG TCA TGA GCC CTT CCA CAA), *Gusb* (Qiagen: QT00176715) *Grina* (fwd: CAA GCC CCT ATG CCT CCC TAT; rev: GGC CCT TGA GGG TAA CCA C), *Xbp1s* (fwd: AAC CAG GAG TTA AGA CAG CGC TT; rev: CTG CAC CTG CTG CGG ACT).

### 4.9. Primary Murine Cortical Cell Culture, Oxygen-glucose-deprivation (OGD) And Cell Viability

Postnatal (P0–P2) primary murine cortical glial cells preparation and seeding was performed as previously described [56,57]. Briefly, after brain dissection meninges and blood vessels were removed, cortex was isolated, homogenized, and dissolved in Dulbecco’s phosphate-buffered saline (DPBS, Pan Biotech #P04-36500, 94501, Aidenbach, Germany) containing 1% (*v*/*v*) trypsin and 0.02% (*v*/*v*) EDTA. The cell suspension was filtered through a 50 μm nylon mesh. After centrifugation (1400 rpm, 5 min), pellets were re-suspended in Gibco™ Dulbeccos’s modified Eagle medium (DMEM, Pan Biotech #P0403550, Germany) and seeded on flasks in DMEM with 10% fetal bovine serum (FBS, Pan Biotech #P30-03306, Germany) and 0.5% penicillin-streptomycin (Pan biotech #P06-07100, Germany). Flasks and plates were coated by poly-L-ornithine (PLO, Sigma-Aldrich #P4957, 64293, Darmstadt, Germany). Cells were kept in a humidified incubator at 37 °C and 5% CO_2_. At passage 2, cells were seeded on experimental plates with phenol red-free Gibco™ Roswell Park Memorial Institute (RPMI 1640, Life Technologies, Carlsbad, CA, USA) with additional 5% FBS and 0.5% penicillin-streptomycin 24 h prior to OGD (Figure 5a). Treatment with the inhibitor of protein glycosylation Tunicamycin (TM) (2 µg/mL) (AppliChem, A2242, Darmstadt, Germany), with the SERCA inhibitor Thapsigargin (TG) (Abcam, Ab120286, Cambridge, UK), PERK inhibitor GSK-2606414 (100 nM) (Calbiochem, 516535, Darmstadt, Germany), IRE1α inhibitor STF-083010 (30 µM) (Axon Medchem, Axon 1670, 9713 GZ Groningen, Netherlands) and treatment with EPO (1 U/mL) (Epoetin alfa Hexal, Hexal, Holzkirchen, Germany) or corresponding controls (NaCl/DMSO) was performed 1 h prior to OGD.

OGD was induced using a self-constructed cube-shaped hypoxia chamber (28 × 14 × 26 cm) with the possibility to measure oxygen, temperature, and pH value in the medium of the cells. The chamber was flooded with pre-warmed (37 °C) inert nitrogen to avoid hypothermia and to replace aerial oxygen as previously described [55,56]. Gas circulation was generated with the help of an inbuilt ventilator. To analyze the OGD-induced cell death, we determined cell vitality using CTB (CellTiter-Blue^®^ assay #G8081, Promega, Mannheim, Germany) and LDH (CytoTox 96^®^ Non-Radioactive Cytotoxicity Assay #G1780, Promega, Mannheim, Germany) according to the manufacturer’s protocol as previously described [55]. Fluorescence or absorption was measured using a microplate reader (Tecan GmbH, Zürich, Switzerland). In both assays, lysed cells (Triton X-100) served as internal positive controls, and viable cells without treatment as negative controls.

### 4.10. Xbp1 mRNA Splicing Assay

IRE1α endoribonuclease activity is involved in the degradation of mRNA substrates and in the unconventional splicing of *Xbp1* mRNA. The latter was monitored using cDNA from brain biopsies and from cells by incubation with *Xbp1* primers to amplify an *Xbp1* amplicon spanning the 26 nt intron from the cDNA samples in a regular 3-step PCR. Thermal cycles were: 5 min at 95 °C, 30 cycles of 30 s at 95 °C, 30 s at 60 °C, and 1 min at 72 °C, followed by 72 °C for 15 min, and a 4 °C hold. PCR was performed using m-XBP1.3 fwd: AAA CAG AGT AGC AGC GCA GAC TGC and h-XBP1.12 rev: TCC TTC TGG GTA GAC CTC TGG GAG primers. PCR products were then digested by PstI resolved by agarose gel (2.5%) electrophoresis and visualized using Midori Green (Biozym Scientific #617004, 31840 Hessisch Oldendorf, Germany) and UV transillumination. The restriction digest of unspliced *Xbp1* (*Xbp1u*) resulted in two fragments of 290 and 183 bp. The size of the *Xbp1s* amplicon lacking PstI sites was 473 bp.

### 4.11. Statistical Analysis

Animals of each genotype were identified by earmark numbers and randomly assigned to the treatment groups by a technical assistant not involved in the analyses. Randomization was carried out using sorting by random numbers (QuickCalcs, Graphpad prism, https://www.graphpad.com/quickcalcs/). All statistical tests were performed using JMP(R), Version 10. SAS Institute Inc., Cary, NC, USA, 1989–2007. Residuals were analysed for normal distribution using the Shapiro-Wilk normality test and variance homogeneity was tested using the Bartlett test. In case of significances in normality and/or variance homogeneity, values were BOX-COX-transformed after calculation of the optimal lambda and used for statistical analysis. Intergroup differences were tested by ANOVA two-way followed by Tukey post-hoc test (multiple groups). Data are given as arithmetic means ± SD. *p* < 0.05 was considered statistically significant. Asterisks indicate significance between group differences, “#” compares EPO vs. vehicle and “§” compares wildtype vs. knock-out genotype. We have demonstrated in a previous study [33] in *Grina^−/−^* mice (81.1 mm^3^ ± 13.8 mm^3^) Infarct sizes twice as large compared to wildtype mice (39.9 mm^3^ ± 10.8 mm^3^). Here, we consider 20% differences in infarct sizes as significant. Therefore, we required a minimum of 4 animals per group to detect such a difference at 95% confidence (*a* = 0.05) and 0.8 power. Power analysis was carried out with G*Power. The test for outliers was not conducted on the data. The individual numbers of animals in our in vivo experiments and the numbers of independent cell culture preparations for in vitro experiments are shown in the legends.

## 5. Conclusions

We have found evidence that the IRE1α and the PERK arm of the UPR are activated in the early phase (6 h) after transient brain ischemia and that the PERK inhibitor GSK-2606414, but not the IRE1α inhibitor STF-083010, reduce the cell death rate and alter Grina transcription. Furthermore, GRINA might play a crucial role in the post-ischemic UPR, since GRINA-deficiency activates the PERK arm of the UPR and increases apoptosis after stroke. Moreover, EPO enhances the post-ischemic activation of pro-survival IRE1α and counteracts the pro-apoptotic PERK branch of the UPR. These findings suggest that both GSK-2606414 and EPO might have therapeutic potential in stroke patients.

## Figures and Tables

**Table 1:** Demographics and haematology of all genotypes and treatment groups after 30 min of tMCAo followed by 6 and 72 h of reperfusion.

Demographics & Haematology												
Genotype	Sham				WT				*Grina^−/−^*			
**Treatment [5000 U/kg bodyweight]**	Vehicle		EPO		Vehicle		EPO		Vehicle		EPO	
**Age [weeks]**	11 (±1)		11 (±1)		11 (±1)		11 (±1)		11 (±1)		11 (±1)	
**Sex [female/male]**	male		male		male		male		male		male	
**Reperfusion time after 30 min of tMCAO**	**6 h**	**72 h**	**6 h**	**72 h**	**6 h**	**72 h**	**6 h**	**72 h**	**6 h**	**72 h**	**6 h**	**72 h**
**Bodyweight [g]**	25.8 (±1.9)	26.7 (±1.0)	26.6 (±1.5)	25.3 (±1.8)	26.5 (±1.9)	25.2 (±2.1)	25.3 (±2.4)	26.4 (±2.0)	26.2 (±1.7)	25.7 (±1.5)	26.4 (±1.7)	27.9 (±1.1)
**WBC [10^3^/µl]**	2.9 (±1.0)	4.0 (±0.9)	4.4 (±1.5)	5.4 (±2.6)	4.1 (±0.6)	1.3 (±0.6)	2.8 (±1.3)	6.3 (±3.5)	6.3 (±1.5)	4.5 (±3.8)	3.9 (±0.4)	3.0 (±1.9)
**RBC [10^6^/µl]**	9.1 (±0.2)	9.3 (±0.1)	9.3 (±0.4)	9.0 (±0.2)	9.2 (±0.6)	10.7 (±0.6)	9.2 (±0.5)	10.4 (±0.4)	9.5 (±1.0)	9.6 (±0.7)	9.2 (±0.3)	9.6 (±0.6)
**HGB [g/dl]**	13.7 (±0.3)	13.6 (±0.1)	13.7 (±0.8)	13.6 (±0.2)	13.8 (±1.1)	16.1 (±1.1)	13.6 (±0.7)	15.7 (±0.3)	12.9 (±0.7)	14.1 (±0.8)	12.8 (±1.2)	14.2 (±1.0)
**HCT [%]**	39.2 (±1.0)	38.8 (±0.7)	39.8 (±1.7)	39.6 (±0.1)	39.6 (±2.7)	44.0 (±2.8)	39.1 (±2.0)	44.7 (±2.0)	40.8 (±3.0)	32.2 (±2.9)	38.8 (±0.9)	40.3 (±2.7)
**MVC [fl]**	42.9 (±0.2)	41.8 (±0.4)	43.0 (±0.2)	44.0 (±1.0)	43.0 (±0.2)	41.6 (±0.2)	42.6 (±0.5)	43.0 (±0.3)	42.9 (±2.1)	40.8 (±0.0)	42.0 (±0.5)	41.8 (±0.8)
**MCH [pg]**	15.0 (±0.2)	14.6 (±0.2)	14.8 (±0.3)	15.1 (±0.6)	15.0 (±0.2)	14.9 (±0.1)	14.9 (±0.3)	15.1 (±0.3)	13.6 (±0.3)	14.7 (±0.2)	13.9 (±1.0)	14.7 (±0.4)
**MCHC [g/dl]**	34.9 (±0.6)	35.0 (±0.9)	34.5 (±0.6)	34.5 (±0.7)	34.8 (±0.4)	35.9 (±0.2)	34.9 (±0.4)	35.1 (±0.9)	31.6 (±3.3)	36.0 (±0.5)	33.0 (±0.9)	35.2 (±0.9)
**PLT [10³/µl]**	720 (±275)	1045 (±61)	916 (±35)	819 (±92)	902 (±113)	1433 (±16)	514 (±507)	966 (±283)	860 (±143)	821 (±47)	1010 (±123)	1153 (±121)

Data are presented in mean ± SD (*n* = 6). Abbreviations. WBC = white blood cells; RBC = red blood cells; HGB = haemoglobin; HCT = haematocrit; MVC = middle corpuscular volume; MCH = middle corpuscular haemoglobin; MCHC = middle corpuscular haemoglobin concentration; PLT = platelets.

## Abbreviations

UPR	Unfolded protein response
IRE1alpha	Inositol-requiring enzyme 1alpha
PERK	Protein kinase RNA-like ER kinase
ATF6	Activating transcription factor 6
GRINA	Ionotropic N-Methyl-D-Aspartate-associated protein
EPO	Erythropoietin
MCAo	Middle cerebral artery occlusion
